# Supplementary material for: HormoneBayes: A novel Bayesian framework for the analysis of pulsatile hormone dynamics
Source: PLoS Comput Biol. 2024 Feb 29;20(2):e1011928. doi: 10.1371/journal.pcbi.1011928 (PMC10931492; doi:10.1371/journal.pcbi.1011928)
Supplement: S1 Text — Fig A: Testing HormoneBayes on synthetic data. Fig B: Assessing the effect of the prior for the LH clearance rate. Fig C: Tuning HormoneBayes when pulses are not clear by using a more informative prior on parameter f. Fig D: Pulse identification using HormoneBayes. Fig E: Using HormoneBayes to identify the effect of interventions on LH pulsatility. (PDF) [file pcbi.1011928.s001.pdf]

Supporting Information

HormoneBayes: a novel Bayesian framework for the analysis of pulsatile hormone dynamics.

Authors: Margaritis Voliotis[1], Ali Abbara[2], Julia K Prague[2,3,4], Johannes D. Veldhuis[5], Waljit S Dhillo[2], Krasimira Tsaneva-Atanasova[1]

1. Department of Mathematics and Living Systems Institute, College of Engineering, Mathematics and Physical Sciences, University of Exeter, Exeter, UK.
2. Department of Metabolism, Digestion and Reproduction, Imperial College London, Hammersmith Hospital, London, UK.
3. Department of Diabetes and Endocrinology, MacLeod Diabetes and Endocrine Centre, Royal Devon and Exeter Hospital, Exeter, UK
4. College of Medicine and Health, University of Exeter, Exeter
5. Emeritus Mayo Clinic, Rochester, Michigan, USA

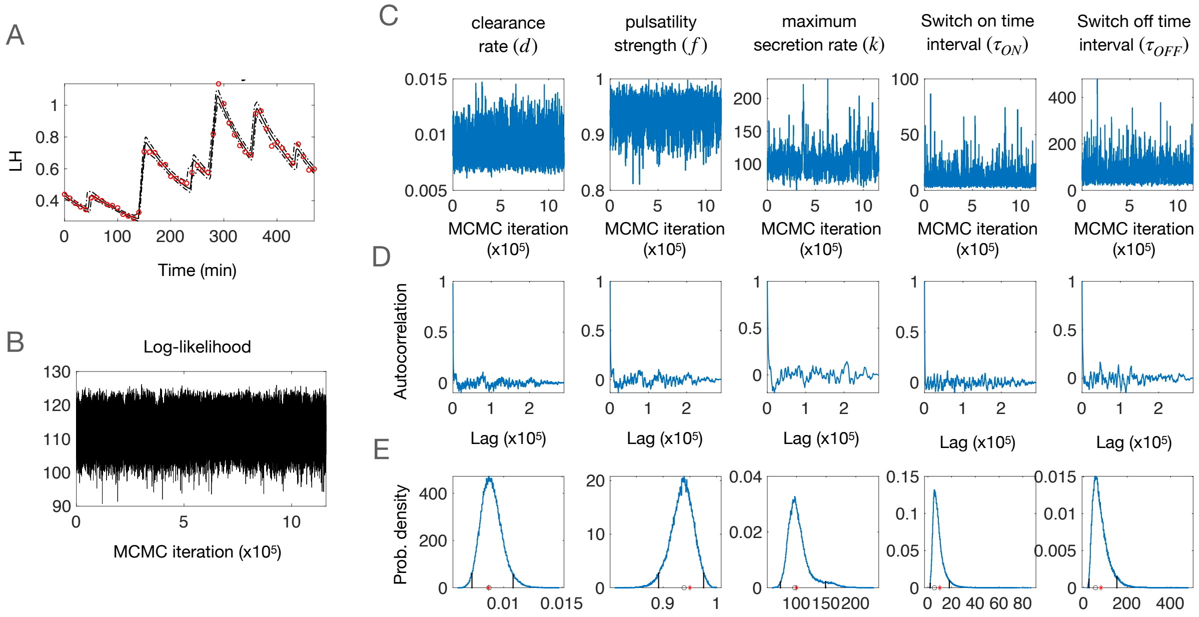

Figure A. Testing HormoneBayes on synthetic data. (A) Synthetic LH data (red circles) along with the model fit generated by HormoneBayes. In this example, synthetic data were generated using the model described in the main text with the following parameter values: $d = 0.0087$, $f = 0.95$, $k = 100$, $\tau_{ON} = 10$, $\tau_{OFF} = 80$. Four independent MCMC chains were generated using the algorithm described in the main text, with each chain consisting of 3 10^5 iterations (the first 10^4 iterations were excluded from further analysis). Traces of the (B) log-likelihood and (C) model parameters. Convergence of the MCMC chains was assessed using their autocorrelation functions (D) and the potential scale reduction factor (R-hat statistic), which in all cases was between 1 and 1.005 indicating good convergence. (E) The posterior parameter distributions accurately reflect the actual parameter values (black asterisks). In each figure, the red circle denotes the maximum a posteriori (MAP) estimate and the vertical black lines the Highest Posterior Density (HPD) credible interval.

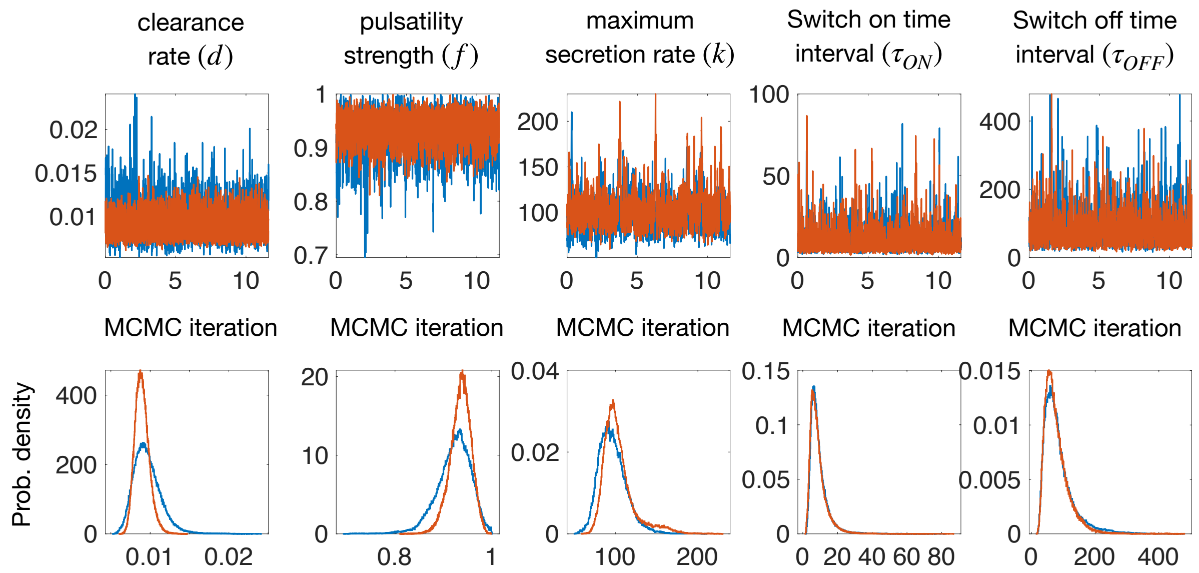

Figure B. Assessing the effect of the prior for the LH clearance rate. We used HormoneBayes to analyse synthetic LH data under two different specifications of the prior for the LH clearance parameter. In the first case (blue) we used an informative prior ($\log(2) \cdot d^{-1} \sim \mathcal{N}(80,9.3)$). In the second case (red) we used an uninformative prior (uniform $\log(2) \cdot d^{-1} \sim \mathcal{N}(10^{-5}, 10^{5})$). In both cases the MCMC chains converged to similar posterior distributions that reflect the parameters used to generate the data. Synthetic data were generated using the model presented in the main text with the following parameters: $d = 0.0087$, $f = 0.95$, $k = 100$, $\tau_{ON} = 10$, $\tau_{OFF} = 80$.

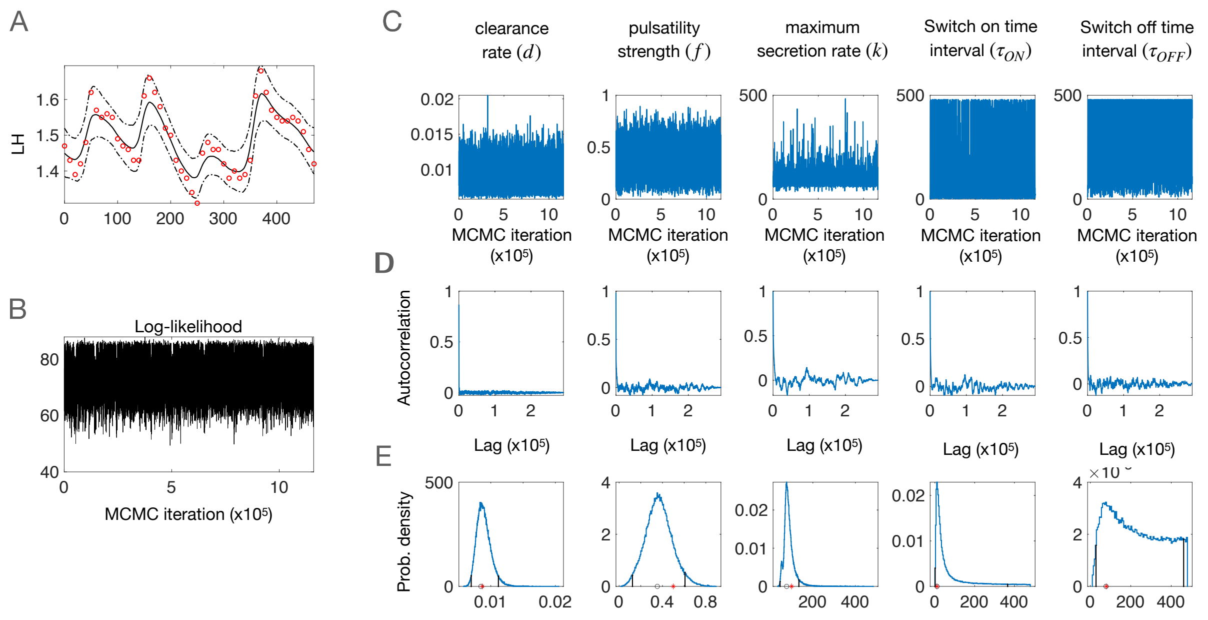

Figure C. Tuning HormoneBayes when pulses are not clear by using a more informative prior on parameter f. (A) Synthetic LH data (red circles) along with the model fit generated by HormoneBayes. In this example, synthetic data were generated using the model described in the main text with the following parameter values: $d = 0.0087$, $f = 0.5$, $k = 100$, $\tau_{ON} = 10$, $\tau_{OFF} = 80$. Here, a Beta distribution with parameters $\alpha = 4$, $\beta = 6$ was used as a prior for parameter f instead of a uniform. Four independent MCMC chains were generated using the algorithm described in the main text, with each chain consisting of 3 10^5 iterations (the first 10^4 iterations were excluded from further analysis). Traces of the (B) log-likelihood and (C) model parameters. Convergence of the MCMC chains was assessed using their autocorrelation functions (D) and the potential scale reduction factor (R-hat statistic), which in all cases was found to be between 1 and 1.009 indicating good convergence. (E) The posterior parameter distributions provide some information about the actual parameter values (black asterisks). In each figure, the red circle denotes the maximum a posteriori (MAP) estimate and the vertical black lines the Highest Posterior Density (HPD) credible interval.

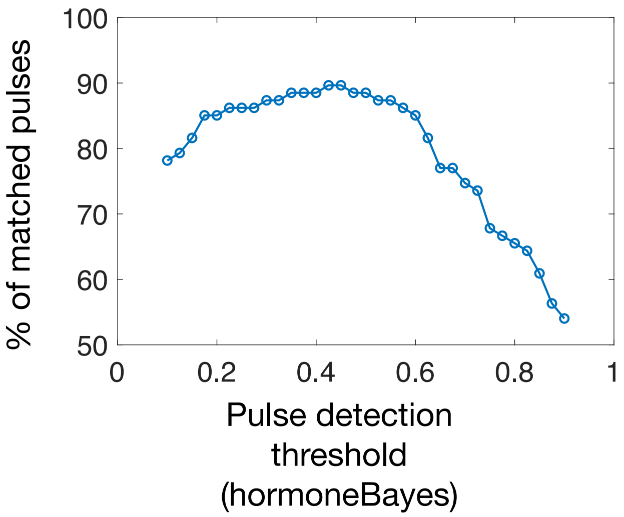

Figure D. Pulse identification using HormoneBayes. Percentage of matched pulses between hormoneBayes and the Deconvolution method as the pulse-detection threshold is varied. Maximum agreement between the two methods is observed when the threshold is around the 0.5 range. For the analysis we used LH data obtained from healthy pre-menopausal women in early follicular phase (n=16).

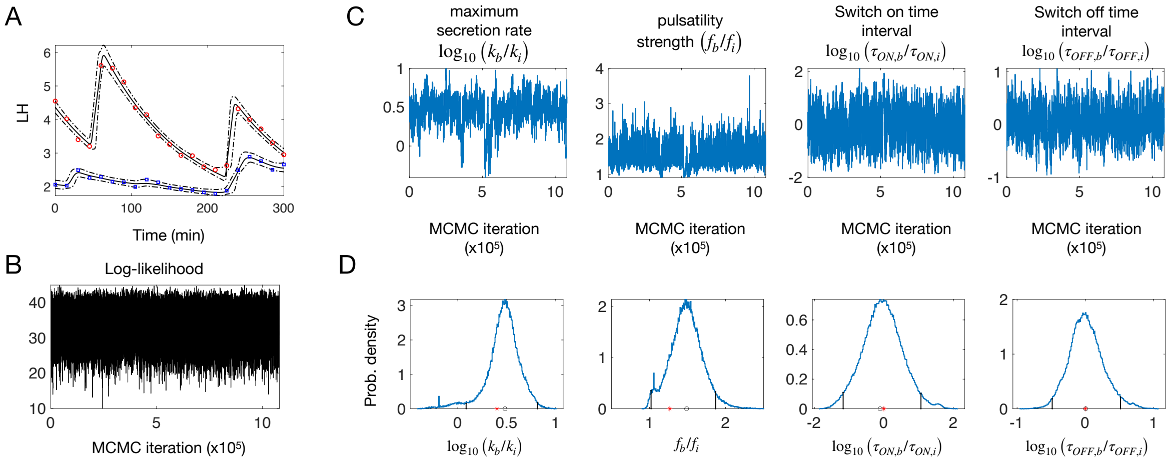

Figure E. Using HormoneBayes to identify the effect of interventions on LH pulsatility. (A) Synthetic LH data from the baseline state (red circles) and the state after a hypothetical intervention (blue squares). Baseline data were generated using the following parameter values: $d = 0.0087$, $f_b = 0.75$, $k_b = 10^{2.4}$, $\tau_{ON,b} = 10$, $\tau_{OFF,b} = 120$. Data corresponding to the state after the intervention were generated by changing the pulsatility strength and maximum secretion rate: $f_i = 0.95$, and $f_i = 10^{2.8}$ (while keeping the other parmeters the same), leading to more pronounced pulses. HormoneBayes was used to fit both datasets and infer the effect of the intervention on the parameters. Traces of the (B) log-likelihood and (C) model parameters. Convergence of the MCMC chains was assessed using the R-hat statistic, which in all cases was between 1 and 1.005 indicating good convergence. (D) The posterior parameter distributions accurately reflect the relationship of parameter before and after the intervention (red asterisks). In each figure, the red circle denotes the maximum a posteriori (MAP) estimate and the vertical black lines the Highest Posterior Density (HPD) credible interval. Four independent MCMC chains were generated, each consisting of 3 10^5 iterations (the first 10^4 iterations were excluded from the analysis).
