# Supplementary material for: HormoneBayes: A novel Bayesian framework for the analysis of pulsatile hormone dynamics
Source: PLoS Comput Biol. 2024 Feb 29;20(2):e1011928. doi: 10.1371/journal.pcbi.1011928 (PMC10931492; doi:10.1371/journal.pcbi.1011928)
Supplement: Attachment — Submitted filename: apppeal_letter.pdf [file pcbi.1011928.s002.pdf]

UNIVERSITY OF EXETER | LIVING SYSTEMS INSTITUTE

Living Systems Institute
University of Exeter
Exeter, EX4 4QD
England, United Kingdom

26 July 2023

Dear Editors,

I am writing to respectfully request a reconsideration of the decision made regarding our manuscript, titled "*HormoneBayes: a novel Bayesian framework for the analysis of pulsatile hormone dynamics*" (PCOMPBIOL-D-23-00942), which was recently rejected to be reviewed by PLOS Computational Biology journal.

We appreciate the time and effort of the Editor in evaluating our work. We understand that the major point raised by the Editor is the supposedly limited applicability of our tool due to the difficulty in obtaining time course data of lutenizing hormone (LH) from patients. Although we acknowledge the difficulty of obtaining LH data from patients in the clinic we would like to emphasise the following points:

1. Assessment of LH dynamics is of paramount importance in clinical research (Prague et al Journal of Clinical Endocrinology and Metabolism 2019; https://doi.org/10.1210/jc.2018-02797)

2. Our method is not limited to human data but can be straightforwardly applied to analyse LH traces collected by researchers studying reproduction in various mammalian models (see for example Clarkson et al PNAS, 2017; https://doi.org/10.1073/pnas.1713897114).

3. Our method can be also applied to assess the pulsatility of other hormonal systems (eg, growth hormone, cortisol)

4. Recently innovative technologies have been proposed in the literature, facilitating real-time monitoring of hormones in the clinic (see Upton et al, Science Translational Medicine 2023, https://doi.org/10.1126/scitranslmed.adg8464; and Liang et al Nature Communications 2019,https://doi.org/10.1038/s41467-019-08799-6). Such technologies further the relevance of tools designed for analysing and interpreting dynamic data.

In the light of these considerations, we are confident that our work makes a valuable contribution to the resources used by endocrinologist and neuroendocrinologists in their study of hormone dynamics. Therefore, we kindly request that you reconsider your decision and allow us to resubmit our work for review.

Thank you very much for considering our appeal. We look forward to your response.

Yours Sincerely,

Margaritis Voliotis, *et al.*
