# Supplementary material for: HormoneBayes: A novel Bayesian framework for the analysis of pulsatile hormone dynamics
Source: PLoS Comput Biol. 2024 Feb 29;20(2):e1011928. doi: 10.1371/journal.pcbi.1011928 (PMC10931492; doi:10.1371/journal.pcbi.1011928)
Supplement: Attachment — Submitted filename: Response to Reviewers.docx [file pcbi.1011928.s003.docx]

We thank the reviewers for their time and effort to review our manuscript. We have implemented all the suggested changes. Below we provide point-by-point answers to the reviewers’ comments. [Line numbers correspond to the manuscript with all changes highlighted].

**Reviewer #1:**

This paper describes the use of Bayesian modelling to provide an alternative to the standard deconvolutional methods used to model hormone pulsatility. When applied to the specific hormone LH, these models provide both a close fit to observed data and useful inferences about the underlying hypothalamus-pituitary modulation. The study demonstrates careful use of parsimonious modelling and high-quality human data, and has results suggesting a clear benefit when compared to the use of purely deconvolutional methods. The ability to compare and contrast pre- and post menopausal women is very interesting, as the literature contains conflicting results in this area.

Major comments:

Novelty is an issue. The users should explain how this work differs from PMID: 14601766 and PMID: 29287490 (the latter also providing open-access Bayesian code for LH pulsatility).

Our approach is distinct to Bayesian adaptations of the deconvolution method presented in PMID: 14601766 and further analysed in PMID: 29287490. In our approach inference is based on a generative model of pulsatile LH secretion with a potential for incorporating mechanistic knowledge of the system. In contrast, deconvolution methods specify a model for a ‘single’ pulse and proceed to describe the data as a ‘mixture’ of independent pulses (which is not true from physiological perspective) occurring at distinct times and hence the number of pulses appears as a parameter to be inferred from the data. In a Bayesian context this means that there is an unknown number of parameters (since the number of pulses is unknown), and hence, the dimension of the posterior is also unknown, making inference a particularly hard problem [Green 1995, <https://doi.org/10.1093/biomet/82.4.711>; Stephens 2000, <https://www.jstor.org/stable/2673981>]. We have now added the reference the reviewer provided and revised the manuscript to make this distinction clearer (lines 96-101 and 240-243).

The results would be improved by (a) an indication of goodness of fit to the data (maybe coefficient of determination added to the upper right subfigure in figure 1) and (b) an example of how a personalised analysis would be performed and used.

We thank the reviewer for their useful suggestions. We have now modified our method to output the value of the log-likelihood, and also report it in the analysis presented in the SI. The log-likelihood can be used as a goodness-of-fit indicator and like the coefficient of determination, which compares the goodness-of-fit of a regression model compared to a null (population average) model, it can be used to compare different models. For instance comparing different models corresponding to different clinical conditions (lines 232-238). As for point (b) we have now included an example (using synthetic data) in the supplementary material that demonstrates how hormoneBayes could be used for personalised assessment of interventions (Fig S5), i.e., to quantify the effect of interventions on the secretion parameters on an individual basis.

“there is a lack of user-friendly methods for computational analysis of the LH data available to clinicians” The current manuscript does not provide enough detail to demonstrate clinician user-friendliness of the distributed code.

Our method comes with a graphical user interface which is already a step change to what is currently available. Nevertheless, we acknowledge that this would require further refinement and improvement, and hence aim to provide regular updates based on user feedback we receive. We have rephased the “user-friendly methods” term throughout the text to make it clear that what we mean is: graphical-user interface software that can be readily downloaded, installed, and used.

Minor comments:  add chmod 755 hormoneBayes (or similar) to the user guide  settings_example.csv (in the user guide) is settings_example.txt (in the distribution)  I get the following error when using the data and settings given in the user guide (mypath is shorthand for my path to the directory containg the executable):  Exception in Tkinter callback Traceback (most recent call last): File "tkinter/__init__.py", line 1892, in __call__ File "pulsatile_GUI.py", line 340, in start_analysis File "shutil.py", line 264, in copyfile FileNotFoundError: [Errno 2] No such file or directory: '/mypath/data/data.csv'

We thank the reviewer for pointing out this bug. The code in the repository has been updated to fix this bug, which was introduced due to an update of the python UI libraries.

"hormoneBayes allows the input of two LH profiling datasets, corresponding to control and perturbed conditions" How is this done? The interface allows only one dataset.

We thank the reviewer for the question. The user still needs to add only one dataset file, in which datasets corresponding to the control and perturbed conditions appear in consecutive lines. We have revised the text (lines 343-353) in the manuscript to explain what we mean, and updated the user manual (<https://git.exeter.ac.uk/mv286/hormonebayes>) to provide specific instruction of how to load such data.

Reviewer #2:

The paper by Voliotis, et al. reports the development of HormoneBayes, a Bayesian model to identify pulsatile hormone dynamics from clinical measurements. The paper would benefit from the following suggestions.

1. Details about the testing of the method are not provided. How was the model applied to the different patients data? Was it the same model or was it different (i.e. different priors) for each type?

Detail information can now be found in the Methods section (lines 336-342). The same model and priors were used across all datasets. Apart from the LH clearance rate (for which reliable measurements exist in the literature) we have used broad, uninformative priors for all other model parameters. We have also used synthetic data to test the effect of a less informative LH clearance prior, and found no significant effect (see supporting information S2)

 2. Details about the gold standard model are not present? What was the method used? How does it differ from HormoneBayes? What are the advantages of HormoneBayes over the existing method – that is, why would someone want to use HormoneBayes and not just use the ‘gold standard’ method? Confidence for the measurement is mentioned as an advantage, but more discussion needs to be added to detail what the differences are and the advantages of HormoneBayes.

The dataset that we have used in our study has been previously analysed using the deconvolution method and the results published (Narayanaswamy et al 2016). These published results are what we are using to validate our method against. We have modified our text to make this clear to the reader (lines 180-183).

As we pointed out in our response to a related comment by reviewer 1, our inference approach is based on a generative model of pulsatile LH secretion, in contrast to the deconvolution approach which models the data as the sum of an unknown number of independent pulses. In a Bayesian context this means that the posterior is of unknown dimensions (as one has an unknown number of parameters) making Bayesian inference particularly hard. We now discuss in detail the difference between the two methods (lines 96-101 and 240-243) as well the advantages of hormoneBayes (see point 5 below)

 3. HormoneBayes seems to produce some false negatives and some false positive results relative to the gold standard methods. The implications of this are not discussed. How impactful would these be on patient care and/or diagnosis?

The deconvolution method has been validated by clinical scientists for its overall soundness and usefulness, but it can still miss or misidentify pulses. As there is no ground truth, the discrepancy between the two methods (albeit small) reflects the uncertainty involved in the pulse identification task (and hence in patient care and diagnosis), and one of the strengths of hormoneBayes is that it provides uncertainty quantification enabling the end-user to access the reliability of the predictions the method makes (lines 233-238).

 4. If possible the gold standard method should be run on the larger dataset and results compared with those of HormoneBayes since this would add to the information about how it performs relative to the standard methods.

We thank the reviewer for the suggestion; however we believe that a detailed comparison goes beyond the scope of this work. Furthermore, it requires a ground truth dataset to make the comparison fair.

 5. The discussion is somewhat limited and should include a recap of the main advantages of HormoneBayes (see point 2), the implications for HormoneBayes – what will it allow that is not currently possible?, a discussion of the practical aspects of HormoneBayes – what is the path for making it to actual use in the clinic?

We thank the reviewer for their helpful suggestions. We have now modified the discussion section to stress: 1) the graphical user interface that makes the method readily accessible to the end-user in the clinic (lines 226-228); 2) the use of a generative model of LH secretion, enabling hormoneBayes to inform and facilitate clinical diagnosis (233-238); 3) the Bayesian nature of the method, enabling personalised analysis (i.e., statistical tests on an individual basis; lines 255-263).

 6. A description of the software is lacking. That is, what language was it written in, what requirements (libraries, etc.) does it have, how does the GUI work, what system(s) is it available on, how easy would it be for someone with limited computational experience to install and use?

We thank the reviewer for these helpful suggestions. All this information can now be found in the README file in the code repository and in the methods section. The core methods have been implemented in C++, while the UI developed in Python (using Tk/Tcl). The only external dependence that the end-user will need to provide is Docker, which can be downloaded free of charge and installed easily for all operating systems. Under the hood, Docker takes care of specific library dependencies making installation and use easy for end-users, regardless of their computational experience.

 7. The Github repository listed appears to have the code available, and the use of a Docker container is welcome, but the documentation is very limited and should be part of the README, not just a Word document. Also, is the software limited to Macintosh only? That seems to be indicated in the Word document.

As pointed out above, the README file in the Git repository has been updated to include relevant information on how to install and use the software (i.e., format of the data, explanation of output files, settings). Currently, the software can be run either on MacOS (intel and M chip) or windows. We also provide .pdf files with step-by-step instructions for installing the software on these operating system.

8. The data described in the paper and on which the results are based does not appear to be available anywhere and should be made available in a suitable fashion (potentially as part of the Github repository) so that the results could be replicated.

The data used in this study have been published and are open access. We have included the links to these publications in the Git repository.
